# Cardiac effects and clinical applications of MG53

**DOI:** 10.1186/s13578-021-00629-x

**Published:** 2021-06-28

**Authors:** Weina Zhong, Dathe Z. Benissan-Messan, Jianjie Ma, Chuanxi Cai, Peter H. U. Lee

**Affiliations:** 1 Department of Surgery, The Ohio State University, Columbus, OH USA; 2 Department of Pathology and Laboratory Medicine, Brown University, Campus Box G-E5, 70 Ship Street, Providence, RI 02912 USA; 3 Department of Cardiothoracic Surgery, Southcoast Health, Fall River, MA USA

**Keywords:** MG53, Heart disease, Membrane repair, Cardioprotection, Diabetes

## Abstract

Heart disease remains the leading cause of mortality globally, so further investigation is required to identify its underlying mechanisms and potential targets for treatment and prevention. Mitsugumin 53 (MG53), also known as TRIM72, is a TRIM family protein that was found to be involved in cell membrane repair and primarily found in striated muscle. Its role in skeletal muscle regeneration and myogenesis has been well documented. However, accumulating evidence suggests that MG53 has a potentially protective role in heart tissue, including in ischemia/reperfusion injury of the heart, cardiomyocyte membrane injury repair, and atrial fibrosis. This review summarizes the regulatory role of MG53 in cardiac tissues, current debates regarding MG53 in diabetes and diabetic cardiomyopathy, as well as highlights potential clinical applications of MG53 in treating cardiac pathologies.

## Introduction

Mitsugumin-53 (MG53), also known as TRIM72, is a cell membrane repair protein that is part of the tripartite motif family of proteins. Similar to other proteins in the TRIM family, MG53 contains the prototypical tripartite motif that includes ring, B-box, and coiled-coil moieties, as well as a SPRY domain at the carboxy terminus [1–3]. It is a striated muscle protein, which is highly expressed in skeletal muscles and to a lesser extent cardiac muscles.

Following acute membrane damage, MG53 senses an oxidized intracellular environment and forms an oxidation-dependent oligomerization repair complex by tethering to phosphatidylserine domains present on intracellular vesicles and in the inner aspect of the plasma membrane [4]. A local elevation of Ca^2+^ enables MG53-tethered intracellular vesicles to fuse with the disrupted plasma membrane, leading to the formation of a repair patch [4]. This process is also facilitated by the interaction of MG53 with muscle specific proteins dysferlin and caveolin-3 (Cav3) [5]. MG53 knock out (KO) mice show progressive myopathy and reduced exercise capacity that is associated with a defect in its membrane repair capability [4].

**Fig. 1:**
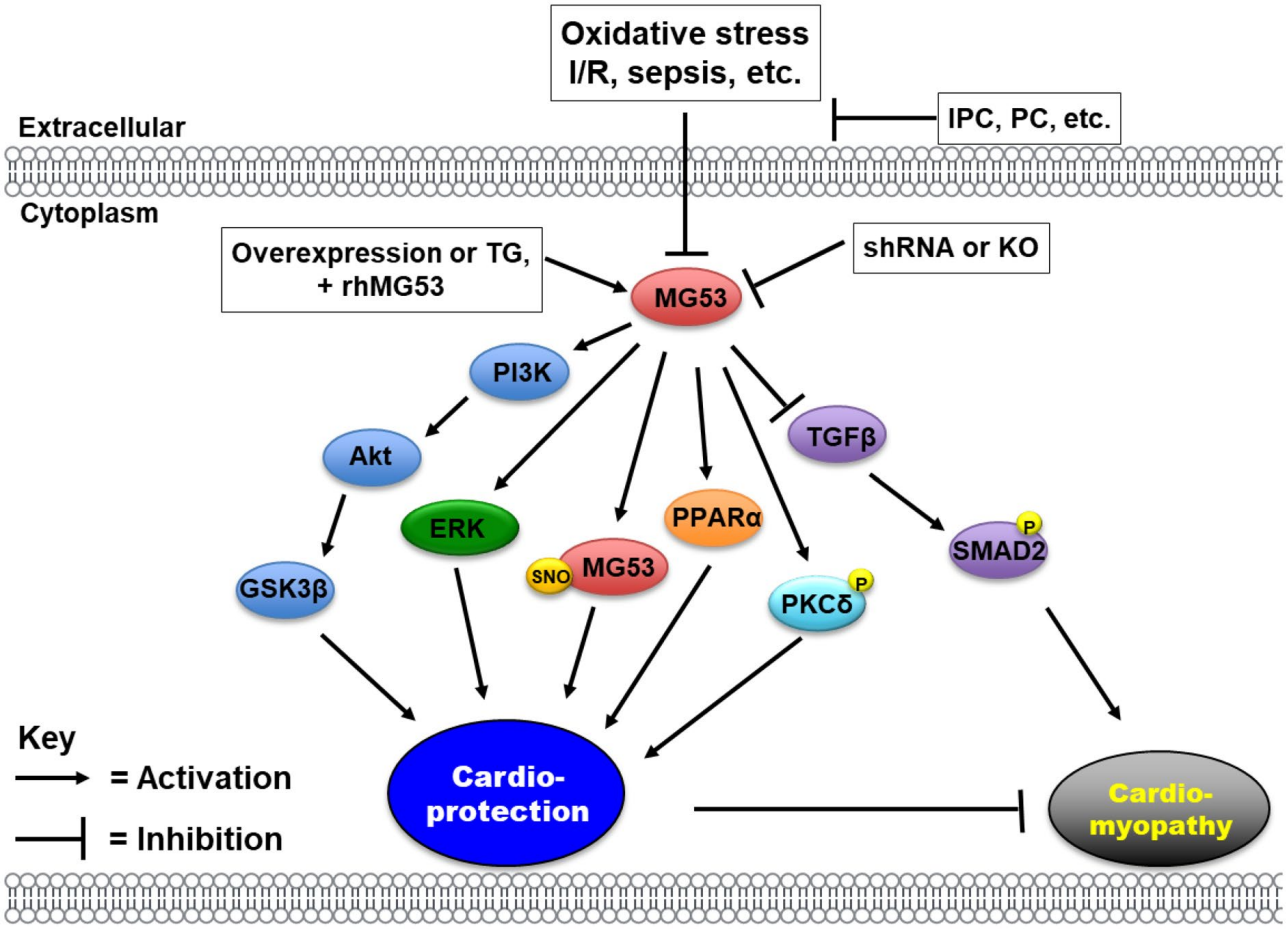
Signal pathways that are associated with the regulatory role of MG53 in heart

In addition to its function in skeletal muscle, MG53 has been shown to have protective effects on various forms of cardiac muscle injury. Since cardiomyocytes are terminally differentiated cells with limited self-renewal capacity, and membrane rupture is a major cause of cardiomyocyte cell death following injury, membrane repair is a necessary process for preserving cardiomyocyte viability [6]. In this review, we summarize the biological function of MG53 with its potential mechanisms in cardiac tissue (Fig. 1), discuss current debates regarding the role of MG53 in diabetic cardiomyopathy (Table 1), and potential clinical applications of recombinant MG53 protein in the management and treatment of heart diseases (Table 2).

**Table 1:** List of controversial studies on the role of MG53 in cardiomyopathy

References	Disease model	Main conclusions	MG53 as a causative factor for cardiomyopathy?	Potential mechanism?
Cao et al. [8]	MG53 KO mice with IPC model	The deficiency of MG53 exhibited myocardial vulnerability to ischemia/reperfusion injury and abolishes IPC protection	NO	Activation of PI3K-Akt-GSK3β pathway
Zhang, et al. [17]	MG53 KO mice with Post-conditioning model	PostC protected wt hearts against IR-induced MI, but failed to protect *mg53-/-* hearts.	NO	Activation of the RISK pathway
Song et al. [32]	HFD, Type II diabetes	Elevated MG53 leads to insulin resistance	YES	E3-ligase-mediated degradation of IRS-1
Ham et al. [61]	αMHC-MG53 TG mice	MG53 TG mice show cardiac hypotrophy at a young age, while exhibit cardiac hypertrophy at older age	Depends on age	E3-ligase-mediated degradation of IRS-1 and the associated Akt signal pathway
Liu et al. [46]	αMHC-MG53 TG mice	Cardiac hypertrophy and cardiomyopathy was induced by the overexpression of MG53.	YES	Regulating PPARα
Liu et al. [62]	MG53 KO mice, TAC	Deficiency of MG53 accelerated pressure overload-induced heart hypertrophy.	NO	Regulate KChIP2 by modulating NF-κB activity
Bian et al. [63]	tPA-MG53 TG mice db/db mice	Mice with sustained elevation of MG53 in their bloodstream show normal glucose handling and lived a healthy lifespan.	NO	Enhanced tissue repair and regeneration
Wu et al. [47]	Diabetic db/db mice	Neutralizing circulating MG53 with monoclonal antibodies has therapeutic effects in *db/db* mice	YES	MG53 binds to the insulin receptor inhibiting the insulin signaling pathway
Wang et al. [60]	db/db mice	Gain or loss of MG53, and administration of rhMG53 did not altered insulin signaling and glucose handling	NO	N/A
Shan et al [18]	IPC, I/R model of mice	IPC induced secretion of MG53 and rhMG53 treatment are cardioprotective against I/R injury.	NO	Elevated activation of PKCδ
Philouze et al. [65]	MG53 KO mice, HFD	MG53 gene knock-down in muscle cells does not lead to impaired insulin response.	NO	N/A

**Table 2:** List of studies for the applications of rhMG53 in the management and treatement of heart diseases

References	Disease model	Dose, route and timing of delivering rhMG53	Functional outcomes	Proposed mechanisms
Han et al. [44]	Sepsis-induced myocardial dysfunction in rats	5 mg/kg; i.v.; 120 min post surgery.	Increased the survival rate with improved cardiac function, and reduced oxidative stress, inflammation, and myocardial apoptosis	Upregulation of PPARα signal pathway
Liu et al. [68]	I/R-induced acute MI in both mice and porcine	1.0 mg/kg; i.v.; prior to ischemia, or 2, 30 min post reperfusion.	Reduced infarct size and troponin I Release, improved cardiac structure and function.	Activation of Akt/GSK3β pathway.
Shan et al. [18]	I/R induced MI w/wo IPC in both mouse and rats	For rats: 3 mg/kg, i.v.; two dose: 5 min prior ischemia and post reperfusion; For mice: 6 mg/kg, i.v.; 40 min before IPC.	Decreased the infarct Size; reduced mortality; decreased cardiac apoptosis, and blood LDH level; Increased LV-EF.	Activation of PKCδ
Wang et al. [60]	Wild type rats	1, 10, and 40 mg/kg; i.v.; every 2 days for a 14-day period	Administration of rhMG53 did not altered insulin signaling and glucose handling	N/A

## Beneficial effects of MG53 in heart disease

### Cardioprotective effects after ischemia/reperfusion injury

Ischemic preconditioning (IPC) was first reported in 1986 by Murry et al. and is an intrinsic process through which repeated short episodes of ischemia are instituted to protect the myocardium against subsequent ischemic insults [7]. Cardiac ischemia is modelled in vitro through the application of hypoxic and oxidative stress. Ischemia/reperfusion (I/R) in mouse hearts and hypoxia/oxidative stress in neonatal rat cardiomyocytes have been associated with a downregulation of MG53. IPC can prevent IR-induced decrease in MG53 expression [8]. MG53 KO mice lack IPC-mediated cardioprotection as evidenced by a failure of IPC to reduce IR-induced myocardial infarct size. IPC suppressed IR-induced infarction in wild type (WT) mouse hearts whereas overexpression of GFP-MG53 fusion protein reduced hypoxia- or H_2_O_2_-induced cell death [8]. However, adenovirus-mediated shRNA targeting MG53 downregulated MG53 expression in rat cardiomyocytes, exacerbating hypoxia-induced cell death and eliminating the protective effect of GFP-MG53 overexpression [8].

IPC activates the reperfusion injury salvage kinase (RISK) and survivor activating factor enhancement (SAFE) pathways to protect the heart against IR injury. The RISK pathway consists of PI3K-Akt-GSK3β and ERK1/2 signaling events (Fig. 1), whereas the SAFE pathway involves activation of tumor necrosis factor-α (TNF-α) and the JAK-STAT3 axis [9–12]. Overexpression of MG53 significantly increased phosphorylation levels of several key pro-survival kinases including Akt, GSK3β, and ERK1/2 over their respective controls [13–16]. However, IPC did not enhance the phosphorylation levels of these key kinases in MG53 KO mouse hearts [8]. This suggests that IPC-induced elevation of PI3K-Akt-GSK3 and ERK1/2 signals is MG53 dependent and that suppression of either pathway fully prevents IPC-induced cardioprotection (Fig. 1). Contrastingly, MG53 has not been demonstrated to activate the SAFE pathway [17].

Interestingly, a recent study from Xiao’s group showed that MG53 is secreted from the mouse heart in response to IPC or oxidative stress, and that the released MG53 protects the heart against IR injury via increased phosphorylation of protein kinase-C-δ (PKCδ) (Fig. 1) [18]. Furthermore, administration of recombinant MG53 proteins to simulate increased circulating MG53 significantly restored IPC function in MG53 KO mice and protected their hearts from IR injury, even without IPC [18].

Postconditioning (PostC) is another form of cardioprotection where the heart is protected against IR injury by brief periods of coronary occlusion at the onset of reperfusion. PostC was shown to protect WT mouse hearts against IR-induced myocardial infarction, myocyte necrosis, and apoptosis, but failed to protect MG53 KO hearts [19]. PostC significantly increased the phosphorylation levels of several important components of the RISK pathway, including Akt, GSK3β, and ERK1/2 in WT mouse hearts but failed to increase the phosphorylation levels of these survival kinases in MG53 KO hearts. MG53 linked to CaV3 and p85 forms the functional complex CaV3-MG53-PI3K, which is essential for MG53-dependent activation of the PI3K-Akt-GSK3β and ERK1/2 signaling pathways (Fig. 1) and leads to MG53-mediated cardioprotection [8]. The activated RISK pathway protects hearts from IR injury [17]. In contrast, dissociation of CaV3 and p85-PI3K in MG53 KO mice led to a failure of PostC-induced cardioprotection [17].

### Membrane repair of cardiomyocytes

In the heart, plasma membrane repair is paramount because cardiomyocytes are terminally differentiated cells and have limited self-renewal capacity [20]. Similar to its role in skeletal muscle, MG53-mediated cardioprotection appears to be through its membrane repair function. In cardiomyocytes, local or global membrane disruption causes MG53 to translocate to the membrane site of injury through a coordinated process that occurs in three steps: (1) sensing of the initial membrane damage in the reduced intracellular environment, (2) formation of a repair complex by oligomerization of MG53, and (3) formation of a repair patch in response to the local elevation in Ca^2+^. Whereas MG53 oxidation confers stability to the repair patch, exposure of membrane cholesterol in the disrupted membrane signals MG53 translocation in a redox-independent manner. The cholesterol-dependent MG53-mediated membrane repair that follows protects the heart from loss of mitochondrial function in stress conditions such as in myocardial IR injury [21].

Evidence suggests that the membrane repair function of MG53 is multifaceted. Repair of plasma membrane damage requires recruitment of intracellular vesicles to the injury site. Overexpression of MG53 increases dysferlin levels and facilitates its trafficking to the muscle membrane [22]. Dysferlin is a muscle specific protein which interacts with Cav3 in the plasma membrane of striated muscle [23]. In 2009, Cai et al*.* demonstrated that MG53 acted as a sensor of oxidation to initiate recruitment of intracellular vesicles to the injury site that could be modulated through a functional interaction with Cav3 [5]. Later, He et al*.* demonstrated that overexpression of MG53 not only increased the quantity of dysferlin and Cav3, but also facilitated their trafficking to and retention at the muscle membrane [22].

MG53-mediated membrane trafficking maintains cell-surface potassium ion current density to ensure the integrity of the action potential profile in cardiomyocytes. Compared to WT mice, the peak potassium ion current density of MG53 KO mouse cardiomyocytes was significantly decreased. Additionally, it was proposed that the activity of voltage-gated K^+^ channel subtype Kv2.1, which is expressed in mouse cardiomyocytes, was specifically increased through MG53-mediated interactions [24]. MG53 KO myocytes retained normal 4AP(4-amin-opyridine)-sensitive currents, but showed significantly impaired 4AP-insensitive currents. Also, the tetraethylammonium chloride (TEA)-sensitive deferred rectifier K^+^ (*I*Kr) currents in MG53-KO myocytes were smaller than in controls. Therefore, the loss of MG53 specifically decreased 4AP-insensitive and TEA-sensitive *I*Kr currents in cardiomyocytes [24].

Zinc deficiency has been associated with pathologies seen in cardiovascular diseases. Zinc is an essential trace factor that plays an important role in wound healing [25]. MG53 contains two zinc-binding motifs [4]. MG53 also interacts with zinc to protect cell membranes against injury [26]. In skeletal muscle, removing extracellular zinc or disrupting the zinc-binding motifs of MG53 alters MG53-mediated vesicular translocation and membrane repair function. The effect of zinc on cell membrane repair was lost in MG53 KO mouse muscle fibers, indicating that MG53 likely serves as a receptor for zinc during cell membrane repair [26].

MG53 may also exert cardioprotective effects through S-nitrosylation (SNO) where it can be S-nitrosylated at cysteine 144 (C144). SNO at C144 protects against oxidation, stabilizes MG53, and promotes cell survival. It can protect MG53 from oxidation-induced degradation and subsequent cell death by shielding C144 from irreversible oxidation [27]. Additionally, irreversible oxidation of MG53 at C144 leads to protein degradation. SNO-MG53 levels have also been shown to be elevated in the myocardium following IPC and PostC.

Besides being a muscle specific membrane repair protein, MG53 has also been shown to be necessary to maintain cardiac transverse(T)-tubule integrity, calcium handling, and cardiac function under pathological cardiac stress. However, MG53 is not required for T-tubule development nor maintenance under normal physiological stress conditions (*i.e*. exercise training) as demonstrated in MG53 KO mice compared to WT littermates [28]. T-tubules form from an invagination of sarcolemma driven by caveolae and/or the addition of new membrane via an action involving exocytosis vesicle trafficking. Additionally, since MG53 plays an important role in vesicle trafficking in response to membrane injury, defective vesicle trafficking and the subsequent loss of membrane repair function may be one of the mechanisms mediating the disruption of the T-tubule structure in MG53 KO mice following cardiac stress [4, 29–31]. The failure of MG53 E3 ligase activity may also mechanistically result in maladaptive T-tubule remodeling [32, 33].

### Regulation of cardiac fibrosis

Atrial fibrillation (AF) is a common arrhythmia that is associated with atrial fibrosis [34]. Atrial fibroblasts are the main determinant of atrial fibrosis [34, 35]. The TGF-β1/Smad pathway plays an important role in relation to atrial fibrosis and AF [36, 37]. The role of MG53 in cardiac fibrosis is still debatable. Studies in human atria suggests a correlation for MG53 expression in atrial fibrosis and AF [38]. However, it is not elucidated whether it is an adapted effect or not since the conclusion was based on the Western blots of whole tissue lysates, including both atrial myocytes and fibroblasts. Rat atrial fibroblasts with depleted MG53 demonstrated reduced expression of TGF-β1, pSmad2, α-SMA, and collagen I, as well as reduced migration and proliferation of fibroblasts [38], indicating MG53 may function upstream of the TGF-β1/Smad pathway to regulate myofibroblast differentiation, migration, and proliferation, and ECM synthesis. The other two in vitro studies also suggested that MG53 positively regulated the proliferation of cardiac fibroblasts [39, 40]. One limitation was observed that neonatal rat cardiac fibroblasts was used in all these in vitro studies [38–40], which might be different with the adult cardiac fibroblasts.

It is still questionable on the role of MG53 in cardiac fibroblasts, as an earlier study by our group showed that there is undetectable expression of MG53 in keratinocytes and fibroblasts [41]. MG53 knockout mice exhibit remarkable defects in skin architecture and collagen overproduction, and display delayed wound healing and abnormal scarring. Furthermore, treatment with rhMG53 protects against acute injury to keratinocytes and facilitates the migration of fibroblasts in response to scratch wounding [41]. Another study from our group showed that rhMG53 can enter valve interstitial cells and suppress transforming growth factor-β-dependent activation of fibrocalcific signaling [42].

Sepsis can cause uncontrolled response of a host’s anti-infective immunity, and lead to severe myocardial dysfunction [43]. In a rat model of septic shock, MG53 expression sharply decreased in the myocardium, which was associated with an increase of oxidative stress and proinflammatory cytokines, and excessive cardiac apoptosis. Treatment with recombinant human MG53 (rhMG53) enhanced both survival rate and cardiac function, reduced the cardiac fibrosis and inflammation, which was associated with PPARα elevation (Fig. 1) [44]. Overall, it would be interesting to reveal the role of MG53 in adult cardiac fibroblasts derived from either human hearts with heart failure/myocarditis, or hearts from diseased animal models in the future.

## Current debates regarding the role of MG53 in diabetic cardiomyopathy

Since the initial discovery of MG53 in 2009 [4, 5, 45], rapid progress has been made in understanding the mechanistic actions of this protein in both the biology of tissue repair and in regulating metabolic syndromes. Several studies published from Xiao and colleagues [32, 46, 47] suggested MG53 might be a causative factor for diabetes (Table 1). Song et al*.* [32] first reported that MG53 expression was significantly upregulated in small animal models of insulin resistance. It was proposed that elevated MG53 might lead to insulin resistance via E3-ligase-mediated degradation of IRS-1. However, the proposed role for MG53-mediated IRS-1 degradation in the metabolic disorders lacks a biological foundation. Although two published manuscripts reported an elevation of MG53 in diabetic rats [48, 49], a greater number of reports [33, 50–55] from multiple independent investigators failed to detect increased MG53 in diabetes and muscle samples derived from human diabetic patients (Table 1). Additionally, mice with insulin resistance showed normal expression of MG53. The IRS family has three other homologous proteins: IRS-2, IRS-3, and IRS-4, all of which contribute to insulin signal transduction. Reports by Terauchi et al*.* and Tamemoto et al*.* showed that knocking out IRS-1 is not sufficient to induce type II diabetes [56, 57]. Also, IRS-3 deficiency does not affect glycemic regulatory capacity [58], indicating the compensatory function among other subtypes of IRS. Only via the knockout of both IRS-1 and IRS-3 does a manifestation of diabetic phenotypes result, indicating that IRS-1 and IRS-3 serve overlapping physiological functions in insulin signaling capabilities [58]. Results from a proteomic study [59] assessed IRS-1 protein interactions in skeletal muscles from normal individuals, obese insulin-resistant nondiabetic control subjects, and patients with type 2 diabetes, before and after insulin infusion. They failed to identify any changes in MG53 protein interaction with IRS-1 across all groups after insulin infusion [59].

Furthermore, a recent published paper from our group [60] revealed lower serum MG53 levels in db/db mice compared with WT littermates (Table 1). Either whole-body knockout of MG53 or sustained increase of MG53 in circulation did not affect insulin signaling and glucose handling in db/db mice. Rats receiving the daily intravenous (IV) treatment of rhMG53 did not have adverse effects on glucose handling [60]. Interestingly, a recent study from Xiao’s group suggested a cardioprotective effect of MG53, where IPC and oxidative stress induced MG53 secretion from the mouse heart via activation of PKCδ and treatment with rhMG53 protected against cardiac IR injury (Table 1) [18]. Therefore, mounting evidence argues against the proposed role of MG53 in diabetes development.

Another study by Liu et al*.* [46] suggested a different role for MG53 regulation of PPARα in cardiomyopathy. αMHC promoter was used to generate transgenic mice (αMHC-MG53) overexpressing MG53 only in heart tissue. It was noted that cardiac hypertrophy and cardiomyopathy was induced by the overexpression of MG53, which may be associated with PPARα-induced lipid toxicity. However, another independent report by Ham et al*.* [61] showed a different cardiac phenotype using the same α-MHC promoter to drive cardiac overexpression of MG53 in mice. They demonstrated that the αMHC-MG53 mice displayed cardio-hypotrophy at younger age when MG53 expression in the heart was already high. It was only when the αMHC-MG53 mice grew older that a hypertrophic heart phenotype was observed (Table 1) [61]. However, even with increased expression of MG53 in the heart, the expression of IRS-1 protein in the αMHC-MG53 mouse heart remained higher than in WT littermates [61], which is contrary to the conclusion that MG53 functions as an E3-ligase to degrade IRS-1 causing the development of diabetes [32]. Another study by Liu and colleagues [62] demonstrated that MG53 deficiency actually accelerated pressure overload-induced heart hypertrophy, indicating an anti-hypertrophic function of MG53 instead. Meanwhile, our group has generated a MG53 transgenic mouse line which achieves sustained elevation of circulating MG53 by fusing a tPA secretory peptide at the amino terminus of MG53 protein (tPA-MG53) (Table 1) [63]. The tPA-MG53 mice maintained sustained elevation of MG53 in their bloodstream and exhibited a healthier lifespan with increased tissue repair and regenerative capability [63].

A separate study recently published in *Circulation* suggested the use anti-MG53 antibody to chelate circulating MG53 in the serum to cure diabetes in db/db mice (Table 1) [47]. Their data indicated that serum level of MG53 is elevated in diabetic animals and in human patients with type 2 diabetes, based on immunoblotting using both commercially available and their custom-made antibodies against MG53. However, a letter to the editor of *Circulation* [64] raised several concerns that may cast doubt on their data interpretation and conclusion. For example, the same commercial antibody (Abcam Cat No. 83302) used by Wu et al*.* indeed detected non-specific bands in serum collected from multiple strains of MG53 WT mice in another recent study from Wang et al*.* [60]. In addition, *Wu *et al*.* showed that the basal level of MG53 in the circulation was in the range of 200–300 pg/ml, which would be outside the range of detection using available immunoblot methods. Results from Wang et al*.* [60] demonstrated that the basal level of MG53 in the WT mice was in the range of 20–40 ng/ml, and the MG53 protein level was indeed significantly diminished in db/db mice compared with WT littermates. Studies were actually performed with a limited number of human diabetic patients and found similar serum levels of MG53 as in healthy volunteers [64]. Additionally, it is failed to observe the association between the circulating level of MG53 and fasting blood glucose in the samples from human patients [60]. These results are dramatically different from those reported by Wu et al*.* [47]. Therefore, care should be taken when concluding the identity and quantity of MG53 in the circulating system.

Another recent independent study from Philouze et al*.* also showed that MG53 is not an essential regulator of insulin signaling and glucose handling via both in vitro and in vivo experiments (Table 1) [65]. Their results revealed that the expression level of MG53 in skeletal muscle is not persistently modulated in various preclinical models of insulin resistance, and knocking down MG53 gene expression in muscle cells does not result in impaired insulin response including Akt phosphorylation and glucose uptake. More importantly, compared to WT mice, both male and female MG53 KO mice still developed high fat-induced obesity and glucose intolerance [65]. Overall, results from multiple independent groups demonstrated that MG53 might not be associated with the diabetic cardiomyopathy.

## Potential clinical applications

Based on the known cardioprotective effects of MG53, rhMG53 protein could potentially be used as a therapeutic protein to prevent, attenuate, or treat cardiac tissue injury from myocardial infarction (MI) or ischemic heart disease. MG53 may also have the potential to serve as a biomarker of cardiac injury.

### Expression of MG53 in human hearts

While it is widely known that MG53 is highly expressed in skeletal muscle, there is debate regarding the degree of MG53 expression in heart tissue. Specifically, there have been inconsistent reports regarding the expression of MG53 in human hearts. Lemckert et al*.* reported that there was no significant physiological expression of MG53 in human hearts [66]. Using three validated antibodies, they demonstrated that MG53 was highly expressed in skeletal and cardiac muscle of mice and rats, but not in human, porcine, or ovine heart samples. More recently, Guo et al*.* demonstrated MG53 expression in human atria through immunohistochemical staining, quantitative PCR, and western blotting [38]. Many different factors may affect the conclusion regarding the expression of MG53 expression in human cardiomyocytes. One limitation is the availability of the specific and sensitive antibodies against human MG53. Recent efforts from our group has developed several epitope specific monoclonal antibodies (mAb) against human and mouse MG53. mAb-914 showed high affinity for MG53 and recognizes MG53 in mouse, pig, sheep, and human hearts with both Western blots and immunofluoresecnt confocal images. Semi-quantitative analysis showed that MG53 protein level in human heart is ~ 1–3% of that in mouse heart. Moreover, through the use of this antibody we found that the expression of MG53 is further decreased in failing human hearts versus non-failing human hearts (unpublished data).

### MG53 as a biomarker of myocardial injury

Marshall et al. attempted to map the proteins released during cardiac myocyte necrosis with the goal to find new molecular components of necrotic injury that could have possible use as biomarkers in clinical [67]. MG53 was found to have a time-dependent relative elevated expression after inducing necrosis by oxidative stress, which supports the potential use of MG53 as a marker for necrotic cellular injury. Using an in vivo murine model, Liu et al*.* demonstrated a low circulating level of MG53 in the blood at baseline which increased in a dose-dependent fashion after an MI [68]. A recent study showed that the serum level of MG53 was elevated in patients with stable cardiovascular disease and reach to the highest level in patients with an acute MI [69]. These findings suggest MG53 may be a potential biomarker of myocardial injury.

### Cardioprotection and therapeutic potential of rhMG53

As discussed above, low levels of endogenous MG53 protein has been found in human hearts, which emphasizes the physiological significance of circulating MG53 for cardioprotection. It has been demonstrated that MG53 can be secreted from skeletal muscle in response to exercise and muscle contraction, and that MG53 circulates in the bloodstream under physiological conditions [70, 71]. Pharmacokinetic (PK) and toxicology assessments support the safety of repetitive IV administration of rhMG53 in Beagle dogs [71]. Studies in mice reported no observable toxic effects with long-term IV or subcutaneous (SQ) administration of rhMG53 [70, 71]. Therefore, therapeutic approaches to modulate MG53 function or systemic administration of rhMG53 protein can potentially act as safe biological interventions to treat cardiac pathologies. As a therapeutic protein, rhMG53 has several practical attributes. First, since native MG53 is present and expressed constitutively in humans and the protein can be found normally in circulation, there are minimal toxicological and immunological concerns for using rhMG53 as a therapeutic protein. Second, rhMG53 can be purified using E. *coli* fermentation, be stored as a lyophilized powder long term at room temperature, and remain soluble and biologically active upon reconstitution [70].

Administration of recombinant human MG53 (rhMG53) mitigated sepsis-induced myocardial dysfunction in rats evidenced by the improved survival rate with increased cardiac function, and reduced oxidative stress, inflammation, and myocardial apoptosis via the elevation of PPARα signal pathway (Table 2) [44]. Given the role of MG53 in IPC and PostC, therapeutic rhMG53 could potentially be used to enhance the protective effects of these maneuvers (Table 2) [18]. However, because IPC can only protect against IR injury before the occurrence of a severe ischemic episode, this limits the practical application of IPC in the clinical setting of an MI [7]. Because PostC is a series of brief ischemia and reperfusion cycles applied after the onset of reperfusion, PostC may have more practical clinic applications [72].

Liu et al*.* used several different animal models to identify whether rhMG53 can play a therapeutic role in treating IR injury. They demonstrated that the administration of rhMG53, either before ischemia or after reperfusion, decreased infarct size in these various in vivo models (Table 2) [68]. Furthermore, they provided evidence demonstrating that rhMG53 preferentially targets infarcted tissue and upregulates phospho-AKT and phosphor-GSK3β supporting the mechanism that rhMG53 concentrates at the site of injury by binding phosphatidylserine [68, 70]. Importantly, animals with the daily administration of rhMG53 did not exhibit side effects on glucose handling (Table 2) [60].

The administration of rhMG53 can come in various forms. The protein can potentially be directly injected at or in the site of myocardial injury to promote repair or attenuate injury. rhMG53 could also be administered by IV, SQ [70], and intramuscular (IM) injections [73]. Exogenously applied rhMG53 has been shown to be able to recognize sites of injury on the membrane of cardiomyocytes and reduce infarct size [68].

There may be potential for gene therapy applications with adeno-associated virus (AAV)-mediated delivery of MG53. For example, given the essential role of MG53 in maintaining the integrity of muscle membranes, MG53 may be used to treat different forms of muscular dystrophy. MG53 gene therapy may also have a role in treating various forms of cardiomyopathies considering the ability of MG53 to maintain the integrity of cardiomyocyte membranes [22]. He et al*.* demonstrated the systemic delivery and muscle-specific overexpression of human MG53 gene by recombinant AAV vectors. They reported enhanced membrane repair and improved muscle and heart function with MG53 overexpression in δ-sarcoglycan-deficient TO-2 hamsters, an animal model of muscular dystrophy and congestive heart failure [22].

## Conclusions

In addition to its function in skeletal muscle, MG53 appears to play an important role in cardiac muscle as well. Its critical function as a membrane repair protein has been clearly demonstrated. It also appears to be important for mediating the cardioprotective effects of both ischemic preconditioning and post-conditioning after ischemia/reperfusion injury. MG53 may also play a role in the development of atrial fibrosis, which, in turn can promote atrial fibrillation. However, there are still debates on the potential role of MG53 in mediating and even promoting diabetes, diet-induced metabolic disorders, and diabetic cardiomyopathies. The potential utility of MG53 as a diagnostic biomarker and rhMG53 as a clinically relevant of therapeutic protein is promising and warrants further study.

## Data Availability

Not applicable.
